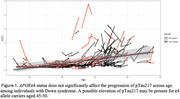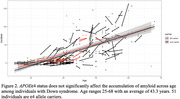# *APOE*ε4 Does not increase rate of amyloid accumulation or tau phosphorylation in adults with Down Syndrome

**DOI:** 10.1002/alz70856_107707

**Published:** 2026-01-09

**Authors:** Omar Abdelmoity, Julie K. Wisch, Benjamin L Handen, Bradley T Christian, Mark Mapstone, H. Diana Rosas, Florence Lai, Joseph H. Lee, Sharon J. Krinsky‐McHale, Frederick A Schmitt, Jordan P. Harp, Christy L. Hom, Ira T. Lott, Sigan L Hartley, Shahid Zaman, Lauren Ptomey, Jeffrey M. Burns, Laura Ibanez, Michael S. Rafii, Elizabeth Head, Beau Ances

**Affiliations:** ^1^ Washington University in St. Louis, St. Louis, MO, USA; ^2^ Washington University in St. Louis School of Medicine, St. Louis, MO, USA; ^3^ University of Pittsburgh, Pittsburgh, PA, USA; ^4^ Waisman Center, University of Wisconsin‐Madison, Madison, WI, USA; ^5^ Department of Medical Physics, University of Wisconsin‐Madison School of Medicine and Public Health, Madison, WI, USA; ^6^ Wisconsin Alzheimer's Disease Research Center, University of Wisconsin School of Medicine and Public Health, Madison, WI, USA; ^7^ University of California, Irvine, Irvine, CA, USA; ^8^ Massachusetts General Hospital, Harvard Medical School, Boston, MA, USA; ^9^ Department of Neurology, Vagelos College of Physicians & Surgeons, Columbia University, New York, NY, USA; ^10^ New York State Institute for Basic Research in Developmental Disabilities, Staten Island, NY, USA; ^11^ University of Kentucky College of Medicine Department of Neurology, Lexington, KY, USA; ^12^ University of Kentucky College of Medicine Sanders‐Brown Center on Aging, Lexington, KY, USA; ^13^ University of Kentucky / Sanders‐Brown Center on Aging, Lexington, KY, USA; ^14^ The UC Irvine Institute for Memory Impairments and Neurological Disorders, Irvine, CA, USA; ^15^ Cambridge Intellectual and Developmental Disabilities Research Group, Department of Psychiatry, University of Cambridge, Douglas House, Cambridge, United Kingdom; ^16^ University of Kansas Medical Center, Kansas City, KS, USA; ^17^ University of Kansas Alzheimer's Disease Center, Kansas City, KS, USA; ^18^ Hope Center for Neurological Disorders, St. Louis, MO, USA; ^19^ Department of Neurology, Washington University School of Medicine, St. Louis, MO, USA; ^20^ NeuroGenomics and Informatics Center, Washington University School of Medicine, St. Louis, MO, USA; ^21^ Alzheimer's Therapeutic Research Institute, Keck School of Medicine, University of Southern California, San Diego, CA, USA; ^22^ Knight Alzheimer Disease Research Center, St. Louis, MO, USA; ^23^ Washington University at St. Louis, St. Louis, MO, USA

## Abstract

**Background:**

Down syndrome (DS) represents a genetic form of Alzheimer's disease (AD) with an earlier expected symptom onset compared to late onset AD (LOAD). It is thought that the extra copy of the Amyloid Precursor Protein (*APP*) gene, located on chromosome 21 contributes to the earlier onset due to increased amyloid deposition in the brain. Hyperphosphorylation of tau protein is also thought to be elevated in the beginning stages of AD pathology within DS. Although *APOE*ε4 has been associated with greater AD risk in LOAD, prior cross‐sectional investigations into the effects of *APOE*ε4 in DS have suggested that there is no additional impact of *APOE*ε4 on the accumulation of amyloid. We aimed to extend this work by examining the associations between longitudinal plasma pTau217 and amyloid PET as a function of *APOE*ε4 status.

**Method:**

Participants with DS were recruited from the Alzheimer's Biomarker Consortium‐Down Syndrome (ABC‐DS) study. Both *p*‐tau217 (*N* = 564 results from 223 individuals including 122 that had 3 results each, Lilly MSD) and Amyloid PET ([11C]‐PiB or [18F]‐AV45) (*N* = 366 scans with 253 unique participants including 113 that had 2 scans each) were acquired. We analyzed the influence ɛ4 allele carrier status had on changes in pTau217 and amyloid across age using linear mixed‐effects modeling, including the age, *APOE*ε4 carrier status and their interaction as covariates. Age was also included as a covariate.

**Result:**

Individuals that are carriers of the *APOE*ε4 allele present with similar baseline amyloid and pTau217 values (*p* = .591 & *p* = .455 respectively). The rate of amyloid and pTau217 accumulation increased across age similarly for both groups (*p* = .772 & *p* = .657 respectively). Although not statistically significant, visual inspection suggests that, with a larger number of participants, individuals between the ages of 45 and 50 who are ɛ4 allele carriers may exhibit elevated pTau217 levels compared to non‐carriers.

**Conclusion:**

We did not observe increased amyloidosis or tau phosphorylation in *APOE*ε4 carriers with DS. Future studies targeting individuals aged 45‐50 are suggested to investigate the potential *APOE*ε4 effect on tau phosphorylation observed in this narrow chronological window, which is close to the average expected age of symptom onset of 52.5 years for DS.